# Sleep and behavioural problems associate with low mood in Finnish children aged 4–12 years: an epidemiological study

**DOI:** 10.1186/s13034-016-0125-4

**Published:** 2016-10-06

**Authors:** K. Maasalo, T. Fontell, J. Wessman, E. T. Aronen

**Affiliations:** 1 Laboratory of Developmental Psychopathology, Helsinki Pediatric Research Center, Children’s Hospital, Child Psychiatry, University of Helsinki and Helsinki University Hospital, Tukholmankatu 8 C 613, 00290 Helsinki, Finland; 2 Children’s Hospital, Child Psychiatry, Helsinki University Hospital, PL 355, 00029 HUS Helsinki, Finland; 3 Laboratory of Developmental Psychopathology, Helsinki Pediatric Research Center, Children’s Hospital, Child Psychiatry, University of Helsinki and Helsinki University Hospital, PL 353, 00029 HUS Helsinki, Finland; 4 Laboratory of Developmental Psychopathology, Helsinki Pediatric Research Center, Children’s Hospital, Child Psychiatry, University of Helsinki and Helsinki University Hospital, Lastenlinnantie 2, 00250 Helsinki, Finland

**Keywords:** Mood, Behaviour problems, Sleep, Child development, Risk factors

## Abstract

**Background:**

Few studies have examined the associations between sleep, mood and behaviour in young children in the early stages of developing psychopathology. The purpose here was to examine the association of emotional problems, especially mood, with family and child characteristics, sleep and behavioural problems in 4–12 year-old children.

**Methods:**

The sample was population-based and included 1714 children. Parents filled in the Strengths and Difficulties Questionnaire and a background questionnaire on child and family characteristics. The associations between (a) emotional symptoms/mood and background variables, (b) emotional problems and conduct problems/hyperactivity and (c) mood and conduct problems/hyperactivity were examined with ordinal regression in univariate and multivariate models.

**Results:**

Of the background variables, child’s sleeping problems had the most powerful association with emotional symptoms and mood. Abnormal emotional problems score and emotional symptoms were associated with abnormal conduct problems and hyperactivity scores. Of the emotional symptoms, low mood was the one associated most strongly with conduct problems and hyperactivity after controlling for all significant background variables and other emotional symptoms.

**Conclusions:**

We conclude that in young children sleep problems associate with low mood. Low mood associates with behavioural problems. When a child presents with low mood or behavioural problems, a comprehensive assessment of their psychiatric symptoms, as well as their sleep problems, is recommended.

## Background

In children, emotional/internalizing problems (e.g. anxiety and fears, low mood) are prevalent [1–3] and precede psychiatric disorders of childhood, adolescence and adulthood [4–6]. Depressive symptoms not meeting the diagnostic criteria of clinical depression have been shown to affect current quality of life and achievements and to predict depression and other psychiatric disorders later in life [2, 7–9]. Approximately half of the young adults disabled due to depression reveal that their psychiatric symptoms emerged already in childhood [10]. Family psychopathology and breakdown, child’s illness and poor socio-economic status have been reported as risk factors for childhood depression [11–14]. Not much is known about the risk factors for specific emotional symptoms, e.g. low mood. In adults, sleep problems predict depressive symptoms and depression [15]. Earlier studies have reported that sleep problems are associated with internalizing and externalizing symptoms in school-aged children [16, 17]. Less is known about the relation between sleep and mood in children [18, 19].

In children, emotional/internalizing symptoms are often associated with behavioural problems in clinical and population-based samples [20–23]. Especially, low or irritated mood is associated with disruptive behaviour [24, 25], raising the question of emerging bipolar disorder in children [26]. The marked increase in diagnosing bipolar disorder in childhood has raised concern about the appropriate classification and treatment of children with persistent irritability. To address this concern, a new diagnostic class of disruptive mood dysregulation disorder has been introduced in the DSM-5 [27]. This new diagnostic entity calls for further research on how mood and behaviour are linked in children in different populations [28]. Population-based studies on the associations between mood and behaviour are especially important as they provide knowledge of these associations at an early stage when confounding factors are limited.

Earlier studies on associations between emotional and behavioural symptoms have mainly examined co-morbidities between disorders meeting the criteria for a distinct diagnosis [20, 22, 29] or assessed the co-morbid problems in larger entities (as internalizing problems, including anxiety and mood and somatic complaints) [21, 30, 31]. Little interest has been paid to individual emotional symptoms and their co-occurrence with conduct problems and hyperactivity, leaving the unanswered question which specific symptoms on the symptom clusters associate the most with behavioural problems. To understand how to better prevent the disorders we need to examine the early stages of these disturbances.

Our main interests in the present study were to examine the child and family characteristics of 4–12 year-old children with emotional problems and low mood in a Finnish population-based sample, and how the emotional problems were associated with sleep, conduct problems and hyperactivity. We were especially interested in how mood was related to conduct problems and hyperactivity in these children.

## Methods

### Sample and procedure

This study is a part of a population-based study on psychiatric problems in children. Statics Finland formed an initial random sample of 5000 Finnish-speaking children covering representative variety of rural and city areas and equal numbers of children from every age group in between 4 and 12 years.

Contact letters were sent to the mothers, except in cases where the civil register knew only information on the father or another caretaker, the contact letter was sent to them.

One parent of each participant filled in a background questionnaire and the Strengths and Difficulties Questionnaire, using either interned-based individual password-protected interface (Digium) or asked for a mailed paper version to fill in. One reminder was sent. The final sample (34.3 %) consisted of 869 girls and 845 boys. Of the children, 668 (39 %) were under school age (4–6 years) and 1046 attended primary school (7–12 years). The sample represented well the original sample with respect to gender distribution and place of residence.

### Questionnaires

The Strengths and Difficulties Questionnaire (SDQ) is a brief 25-item instrument to screen the emotional and behavioural problems of children and adolescents [32]. The items are categorized into five subscales (subscores ranging from 0 to 10): emotional problems, conduct problems, hyperactivity, peer problems and prosocial scale. The SDQ has been shown in epidemiological studies [33] to be applicable to Finnish children. Of the items screening for emotional problems, “often unhappy, down-hearted or tearful” is the one directly describing mood, while the others describe anxiety symptoms and somatic complaints. The mood item (no. 13) was used as a measure for mood in the sample.

Background information was collected from parents with a questionnaire covering child’s age, gender, number and ages of siblings, parents’ marital status, people living with child, parents’ job, parents’ education level, health issues of the child and whether the child needs any support in day care or school. A brief question of current sleeping problems was also included (“Does child have a sleeping problem: yes/no”).

### Statistical analyses

For statistical purposes, new variables were formed from the background information collected to avoid overlapping and high correlations between the variables. The information on parents’ marital status and people living with child were combined to form a variable “biological parents living with child” with categories “both” (82.5 %), “only mother” (16.0 %) and “only father” (1.3 %). The variable describing family’s socio-economic status (higher SES of the parents living with child) was combined from the parents’ jobs (retired and students excluded due to their small numbers; correlation between mothers’ and fathers’ jobs r_s_ = 0.399, N = 1639, p < 0.001, 2-tailed)). Educational level was excluded due to its correlation with parents’ jobs (mothers: r_s_ = 0.427, N 1663, p < 0.001, 2-tailed, fathers: r_s_ = 0.498, N = 1675, p < 0.001, 2-tailed). Child’s need of special support was excluded as it correlated with a child having an illness or disability (r_s_ = 0.306, N = 1696, p < 0.001, 2-tailed).

The SDQ subscores were categorized as “normal”, “borderline” or “abnormal” using the cut-off points defined in the official SDQ website [34] (0–3; 4; 5–10 for emotional problems, 0–2; 3; 4–10 for conduct problems and 0–5; 6; 7–10 for hyperactive problems, respectively). The “somewhat true” and “certainly true” categories of the emotional symptoms were collapsed into a “somewhat or certainly true” category in order to achieve more statistical power.

The associations between (a) background variables and emotional symptoms/mood, (b) emotional problems scores and conduct problems/hyperactivity scores and (c) emotional symptoms and conduct problem/hyperactivity scores were examined with ordinal regression in univariate and multivariate models.

Analyses were carried out using IBM SPSS Statistics 22.

## Results

### Descriptive statistics and family and child characteristics related to emotional problems and mood

The characteristics of the children and their families are presented in Table 1. In the SDQ, boys had higher total difficulties scores and all other problem subscale scores than girls, except the emotional problems subscale score, where no difference existed between the genders. The emotional problems score increased with increasing age, while the other problem scores decreased (ordinal regression, problem scores and age as continuous variables, p’s 0.000–0.006). Table 2 presents the proportions of children scoring normal/borderline/abnormal on SDQ subscale scores and not true/somewhat true/certainly true on emotional symptoms subscale items. Emotional problems score was abnormal in 5.8 % of the children, and low mood (defined as SDQ item 13 being somewhat or certainly true) was reported in 16.0 % of the children.

**Table 1:** Descriptive statistics (n = 1714)

	n (%)	
Age in years, mean (SD)	7.9 (2.3)	
Preschool age	668 (39.0)	
School age	1046 (61.0)	
Girls	869 (50.7)	
Boys	845 (49.3)	
Sleeping problems	246 (14.4)	
Illness or disability	262 (15.3)	
Living in same household		
Mother	1688 (98.5)	
Father	1436 (83.8)	
Siblings	1454 (84.8)	
Stepmother	10 (0.6)	
Stepfather	102 (6.0)	
Grandparent	6 (0.5)	
Residential area		
Major city	659 (38.4)	
Medium-sized city	737 (43.0)	
Small city/town	316 (18.4)	
Unavailable information	1 (0.1)	
Parents’ marital status		
Married	1159 (67.6)	
Divorced/live separately	268 (15.6)	
Common-law marriage	259 (15.1)	
Some other	23 (1.3)	
Unavailable information	5 (0.3)	

	Mother n (%)	Father n (%)
Parents’ occupation		
Manager	32 (1.9)	136 (7.9)
Managerial employee	314 (18.3)	401 (23.4)
White collar worker	487 (28.4)	264 (15.4)
Entrepreneur	109 (6.4)	292 (17.0)
Blue collar worker	666 (38.9)	563 (32.8)
Student	52 (3.0)	7 (0.4)
Retired	5 (0.3)	17 (1.0)
Unavailable information	49 (2.9)	34 (2.0)

**Table 2:** Proportions of scores on SDQ subscales and on emotional problems subscale items

SDQ scores	Normal n (%)	Borderline n (%)	Abnormal n (%)
Total difficulties score	1529 (89.2)	70 (4.1)	115 (6.7)
Emotional problems score	1517 (88.5)	97 (5.7)	100 (5.8)
Conduct problems score	1334 (77.8)	184 (10.7)	196 (11.4)
Hyperactivity score	1534 (89.5)	69 (4.0)	111 (6.5)
Peer problems score	1334 (77.8)	193 (11.3)	187 (10.9)
Prosocial score	1429 (83.4)	178 (10.4)	107 (6.2)

Items in emotional problems subscale	Not true n (%)	Somewhat true n (%)	Certainly true n (%)
Often complains of headaches, stomach aches or sickness	1109 (6.7)	518 (30.2)	87 (5.1)
Many worries, often seems worried	1351 (78.8)	325 (19.0)	38 (2.2)
Often unhappy, down-hearted or tearful	1441 (84.1)	253 (14.8)	20 (1.2)
Nervous or clingy in new situations, easily loses confidence	940 (54.8)	657 (38.3)	117 (6.8)
Many fears, easily scared	1470 (85.8)	215 (12.5)	29 (1.7)

Table 3 presents the results of univariate and multivariate ordinal regression on how the family and child characteristics relate to emotional problems and mood. Having sleeping problems had the strongest association with emotional problems and low mood.

**Table 3:** Relationships between child and family characteristics, emotional problems score and mood

	Emotional problems score		Often unhappy, down-hearted or tearful	
	Univariate	Multivariate	Univariate	Multivariate
	OR (95 % CI)	OR (95 % CI)	OR (95 % CI)	OR (95 % CI)
Age in years	1.11 (1.04–1.19)*	1.08 (1.01–1.15)*	1.09 (1.03–1.15)**	1.06 (1.00–1.12)
Gender: girl	1.12 (0.83–1.50)	1.28 (0.94–1.76)	0.98 (0.75–1.26)	1.04 (0.79–1.36)
Biological parents living with child				
Both	0	0	0	0
Mother only	2.61 (1.86–3.67)***	1.99 (1.37–2.89)***	2.26 (1.66–3.07)***	1.88 (1.36–2.60)***
Father only	2.86 (1.11–7.34)*	2.77 (1.01–7.65)*	2.98 (1.27–7.00)**	3.00 (1.23–7.29)*
Child has siblings	0.71 (0.48–1.03)	0.84 (0.55–1.26)	0.81 (0.57–1.14)	–
Higher SES of parents living with child				
Manager	0	–	0	–
Managerial employee	0.75 (0.42–1.33)	–	0.96 (0.56–1.65)	–
White collar worker	0.82 (0.46–1.46)	–	1.19 (0.69–2.03)	–
Entrepreneur	0.67 (0.32–1.37)	–	1.20 (0.64–2.22)	–
Blue collar worker	1.27 (0.72–2.22)	–	1.53 (0.90–2.61)	–
Child has illness or disability	3.56 (2.56–4.94)***	2.84 (2.00–4.02)***	2.66 (1.96–3.62)***	2.21 (1.61–3.05)***
Child has sleeping problems	5.19 (3.75–7.19)***	4.31 (3.07–6.06)***	3.18 (2.34–4.33)***	2.70 (1.96–3.72)***

Regression analysis examining emotional problems and mood as outcome variables and child and family characteristics as predictor variables. The emotional problems score was categorized as normal, borderline or abnormal (0–3; 4; 5–10 respectively) and the mood item dichotomized as “not true” and “somewhat/certainly true”

*OR* odds ratio; *CI* confidence interval

* p < 0.05, ** p < 0.01, *** p < 0.001

### Relationship between emotional problems/mood and conduct problems

A significant association emerged between scores for emotional problems and conduct problems. Children with a borderline emotional problems score had over three-fold odds for having a conduct problems score within the abnormal range (OR 3.25, 95 % CI 2.10–5.04). Children with an emotional problems score within the abnormal range had nearly eight-fold odds for scoring within the abnormal range on conduct problems than those with an emotional problems score within the normal range (OR 7.86, 95 % CI 5.20–11.88). We controlled for age, gender and family SES.

In univariate analysis, after controlling for age, gender and family SES, all emotional symptoms were associated with conduct problems. The results are presented in Table 4. The strongest association was found between mood and conduct problems. We also looked at the model including an interaction term for mood and sleep, but no statistically significant interaction was found (data not shown). In multivariate analysis, low mood remained as the most powerful association with conduct problems. After adding all of the other child and family characteristics related to problems (in addition to age, gender and SES, also illness/disability and sleep problem of the child and absence of the other biological parent in the family) to the multivariate analysis, mood, somatic complaints and worrying remained significantly associated with conduct problems (OR 1.51–3.19), mood remaining as the strongest association with conduct problems (OR 3.19, 95 % CI 2.26–4.49).

**Table 4:** Emotional symptoms, conduct problems and hyperactivity

	Conduct problems score		Hyperactive score	
	Univariate	Multivariate	Univariate	Multivariate
	OR (95 % CI)	OR (95 % CI)	OR (95 % CI)	OR (95 % CI)
Often unhappy, down-hearted or tearful	5.64 (4.26–7.45)***	3.33 (2.38–4.67)***	4.42 (3.13–6.24)***	2.78 (1.80–4.28)***
Often complains of headaches, stomach aches or sickness	2.36 (1.85–3.03)***	1.61 (1.23–2.10)***	2.01 (1.45–2.79)***	1.33 (0.93–1.90)
Many worries, often seems worried	3.86 (2.95–5.05)***	1.70 (1.22–2.37)**	3.03 (2.15–4.27)***	1.32 (0.85–2.05)
Nervous or clingy in new situations, easily loses confidence	1.43 (1.13–1.81)**	1.05 (0.81–1.36)	1.54 (1.12–2.12)**	1.10 (0.78–1.55)
Many fears, easily scared	3.06 (2.29–4.10)***	1.43 (1.01–2.02)*	3.42 (2.38–4.90)***	1.88 (1.23–2.88)*

Regression analysis examining the problem scores as explained variables and the emotional symptoms as predictor variables. The problem scores were categorized as normal, borderline or abnormal (0–2; 3; 4–10 for conduct problems and 0–5; 6; 7–10 for hyperactive problems, respectively) and the emotional symptoms dichotomized in “not true” and “somewhat/certainly true”

* p < 0.05, ** p < 0.01, *** p < 0.001

*OR* odds ratio; *CI* confidence interval

### Relationship between emotional problems/mood and hyperactivity

A significant association was present between emotional problems scores and hyperactivity scores. Children with borderline emotional problems scores had almost three-fold odds for having a hyperactivity score within the abnormal range (OR 2.90, 95 % CI 1.66–5.01) than those with an emotional problems score within the normal range. Children with an emotional problems score within the abnormal range had nearly eight-fold odds for scoring within the abnormal range on hyperactivity (OR 7.73, 95 % CI 4.86–12.28) than those with a normal-range emotional problems score. We controlled for age, gender and family SES.

As with conduct problems, in univariate analysis, all of the emotional symptoms were associated with hyperactivity, after controlling for age, gender and family SES. The results are presented in Table 4. The strongest association between single emotional symptoms and hyperactivity was found for mood and hyperactivity. Again, we looked at the model including an interaction term for mood and sleep, but no statistically significant interaction was found (data not shown). In multivariate analysis, mood remained as the most powerful association with hyperactivity. Again, after adding all of the other child and family characteristics related to problems to the multivariate analysis, only mood remained significantly associated with hyperactivity (OR 2.50, 95 % CI 1.60–3.91).

### Associations between mood, conduct problems and hyperactivity

Of all the children with low mood (n = 273), 13.1 % scored within the abnormal range on both conduct problems and hyperactivity scales, compared to 2.2 % of the children without low mood. Of the 68 children (4.0 % of the whole sample) scoring within the abnormal range in both conduct problems and hyperactivity scales, slightly over half also showed low mood (n = 36, 2.1 % of the whole sample). See also Table 5.

**Table 5:** Mood and conduct problems/hyperactivity

	Often unhappy, down-hearted or tearful	
	Not true, n (%)^a^	Somewhat or certainly true, n (%)^a^
Conduct problems score		
Normal range	1197 (89.7)	137 (10.3)
Borderline	138 (75.0)	46 (25.0)
Abnormal range	106 (54.1)	90 (45.9)
Hyperactive score		
Normal range	1331 (86.8)	203 (13.2)
Borderline	49 (71.0)	20 (29.0)
Abnormal range	61 (55.0)	50 (45.0)
Both conduct problems score and hyperactivity score within abnormal range	32 (47.1)	36 (52.9)

^a^ % of children within the same range of conduct problems/hyperactivity scores

## Discussion

We aimed to examine the characteristics of 4–12 year-old children with emotional problems and low mood in a Finnish population-based sample. Our main interest was in evaluating how emotional problems, especially mood, were associated with conduct problems and hyperactivity.

In our sample, 5.8 % of the children scored within the abnormal range on emotional problems in the parent-rated SDQ. This proportion is in line with an earlier Finnish study investigating the frequencies of psychiatric symptoms in 8–9 year-old children using different methods, which reported that 5.5 % of the children had a probable emotional disturbance [35], or a Danish study in a community sample of 12–14 year-old children reporting a frequency of 6.6 % having an abnormal emotional subscale score in the SDQ [31]. Much higher frequencies have also been reported [30, 36]. The frequency of low mood in our study was 16.0 and 1.2 % of the children were reported as being often unhappy, down-hearted or tearful. Low mood is not the same as depression, but since persistent low mood is one of the core symptoms of depression, comparing the two may be justified. The prevalence of major depression in children is estimated to be 2 %, and subthreshold depressive symptoms are found in 10 % of children and adolescents [37] (point prevalence estimates ranging from 0.03 to 7 % for major depression and from 0.24 to 14 % for subthreshold depression [2]).

Characteristics associated with both emotional problems and low mood in our sample were not living with both biological parents, having an illness or disability and having sleeping problems. Interestingly, sleeping problems were an even stronger associate of emotional problems and low mood than family structure or child’s illness. After controlling for other risk factors, sleeping problems remained the strongest associate of low mood. Earlier studies have reported strong associations between sleep problems and internalizing and externalizing symptoms in school-aged children [16, 17]. Our study adds to this knowledge by showing a strong association between sleep problems and low mood in a population-based sample of children. Because of the cross-sectional nature of the study, the causal direction cannot be determined. However, based on earlier studies there is more support for the pattern that sleep disruption precedes and possibly causes depression [38, 39]. Earlier data also suggest that the association of children’s sleep problems with mental health issues could be mediated by disruptions of daily mood [19]. These suggestions and the results of our study highlight the importance of recognizing and treating sleep problems in children. Somatic illness has earlier been shown to be associated with psychosocial problems, especially emotional symptoms [12, 40, 41]. Our result supports these findings and stresses the need to pay attention to the emotional well-being of small children with illness or disability.

Scores on the emotional symptoms scale were associated with conduct problems and hyperactivity. Similar associations have been reported in studies with differing methods [14, 21, 22]. Interestingly, we found that of the single emotional/internalizing symptoms, low mood was the strongest associate of conduct problems and hyperactivity. Even after controlling for the other emotional symptoms and all significant background variables, the mood item remained highly significantly associated with conduct problems and hyperactivity scores. This may suggest that there is a common underlying factor in mood and behavioural problems. Children with simultaneous problems with mood and behavioural control might be the ones at risk of developing bipolar disorder (Uchida et al. [26]).

Previous studies in population-based and clinical samples of children have mainly investigated co-morbidities of diagnosed disorders; we found no studies of comparable data of associations at symptom level. Our findings are, however, in line with the established facts that depression and disruptive behaviour are often co-morbid [20, 29, 42]. Angold and colleagues (1999) published a meta-analysis of 21 community-based studies examining psychiatric co-morbidities, reporting increased odds for co-morbid depression and conduct disorder, depression and attention-deficit/hyperactivity disorder (ADHD), and ADHD and conduct disorder. Only one included study was on children aged 7–11 years, while the majority of samples consisted of pre-adolescents or adolescents. Thus, our study with 4–12 year-old children complements the existing data and provides valuable information on the early stages of psychopathology.

Of the children in the present study, 2.1 % were reported as simultaneously having low mood, conduct problems and hyperactivity. In view of what has been published on the effect of subsyndromal depressive symptoms and co-morbid depression, conduct disorder and ADHD, this is a notable subgroup of children in a population sample, as this combination of symptoms is likely to affect how these children cope in everyday life and to predict future psychopathology [2, 23, 43].

One of the most intriguing findings of our study was mood standing out from the other emotional symptoms associating with both conduct problems and hyperactivity. Our finding might suggest that low mood accounts for the associations between emotional symptoms and conduct problems/hyperactivity more than other emotional symptoms. Longitudinally, depression is suggested to usually be the consequence of another psychiatric disorder, with the exception of conduct disorder, which might in fact result from depression [37, 44]. Our finding may suggest that among children with emotional problems, children presenting with low mood are at special risk for conduct problems and hyperactivity.

Our sample represented well the originally selected sample with respect to gender distribution and place of residence, but the rather low rate of participation remains a possible source of bias. Because the data are cross-sectional, we can make observations on the concurrence of symptoms and associated characteristics, but cannot draw conclusions on causality. All data were provided by parents. We did not ask the children about their problems, and there was no clinical evaluation to set diagnosis. Internalizing problems are known to be reported less by parents than by children [33, 45]. It is thus possible that the prevalence of emotional problems is actually higher than presented here. Using only a single rater, the observed associations may in part reflect the fact that a single rater might be prone to report co-occurrence of problems. However, as about half of the children were under school age, it was not possible for them to fill in the questionnaires. In many population-based studies, the parental report remains the only information source on a child’s symptoms.

## Conclusions

This study adds to the research suggesting interplay between sleep problems, mood and behaviour in 4–12 year-old children. The findings highlight the associations between sleep problems and mood and between mood and problematic behaviour. When a child presents with low mood or behavioural problems, a comprehensive assessment of their psychiatric symptoms, as well as their sleep problems, is recommended. Follow-up studies are needed to examine the causal relationship.

## Abbreviations

ADHD: attention-deficit/hyperactivity disorder
SDQ: Strengths and Difficulties Questionnaire
SES: socio-economic status
